# Supplementary material for: Deciphering the Tangible Spatio-Temporal Spread of a 25-Year Tuberculosis Outbreak Boosted by Social Determinants
Source: Microbiol Spectr. 2023 Feb 14;11(2):e02826-22. doi: 10.1128/spectrum.02826-22 (PMC10100973; doi:10.1128/spectrum.02826-22)
Supplement: Supplemental file 1 — Supplemental material. Download spectrum.02826-22-s0001.pdf, PDF file, 0.8 MB [file spectrum.02826-22-s0001.pdf]

Supplemental Information

Deciphering the tangible spatio-temporal spread of a 25-year tuberculosis outbreak boosted by social determinants

Mariana G. López[1], Mª Isolina Campos-Herrero[2], Manuela Torres-Puente[1], Fernando Cañas[3], Jessica Comín[4], Rodolfo Copado[5], Penelope Wintringer[6], Zamin Iqbal[6], Eduardo Lagarejos[2], Miguel Moreno-Molina[1], Laura Pérez-Lago[7], Berta Pino[8], Laura Sante[9], Darío García de Viedma[7,10,¶], Sofía Samper[4,10,¶,*], Iñaki Comas[1,11*]

Epidemiological context of the Canary Islands

Gran Canaria, the most populated island in the archipelago, displayed a decreasing TB incidence from 32.2 cases per 100,000 inhabitants in 1988 to 29 in 1992, the year before the outbreak onset. The first years of the outbreak had an incidence of around 29 cases per 100,000 inhabitants (1–4). Meanwhile, the prevalence of Beijing (L2) strains increased from 5.5% of total cases in 1993 to 27.1% in 1999 (5), with a prevalence of 20.9% reported in 2014 (6). Alcoholism was the most important risk factor associated with TB before 1993, accounting for ~35% of total cases (2). During the first years of the outbreak (1993-1999), the only risk factor reported was HIV infection, accounting for 14% of cases (3). In 2000, the TB surveillance program was implemented in the Canary Islands by the Canarian Public Health Service, which prompted a reduction in TB incidence from 23.4 in 2000 to 6.3 in 2019 (https://www3.gobiernodecanarias.org/sanidad/scs/listaImagenes.jsp?idDocument=fe9e3e94-feee-11e0-ab85-376c664a882a&idCarpeta=b25ca6dc-a9a4-11dd-b574-dd4e320f085c).

Study design

Canarian Hospitals performed TB diagnosis (GeneXpert and culture). The first cases were sent to the Instituto Aragonés de Ciencias de la Salud (Zaragoza, Spain), where they were typified by molecular methods (1). After the outbreak was declared, DNA extraction and RFLP typing were conducted in the Hospital Universitario de Gran Canaria Dr. Negrín between 1993-1996 (2). In the following years, inactivated samples from Canarian Hospitals were sent to the Instituto Aragonés de Ciencias de la Salud, for outbreak surveillance using spoligotyping. Epidemiological data was obtained retrospectively from the different Canarian Hospitals, Madrid, Zaragoza and Valencia.

Temporal signal analysis

Before applying a phylodynamic approach to study the GC outbreak's evolution, our

dataset's clock signal was evaluated and compared with previous TB outbreaks. A moderate but significant clocklike structure was obtained with TempEst analysis (R^2=0.32, p-value<0.0001), which was stronger in comparison with outbreaks from Denmark (R^2=0.23, p-value<0.0001)(3); Switzerland (R^2=0.05, p-value<0.0001)(4); Thailand (R^2=0.14, p-value = 0.011)(5), and Argentina (R^2=0.08, p-value<0.0001)(6) (Figure S2). Date randomization test (DRT) was also performed, which involves randomly reassigning sampling times of sequences. DRT was repeated 100 times, and the same BDSKY analysis described in the methods was performed for each replicate. The simple DTR was passed since the observed data's median clock rate value does not overlap with the 95% HPD (highest posterior density interval) from the randomized test (Figure S3). Only one randomized replicate overlapping with observed data occurred -this was the result of date similarity among samples, - and this replicate is very similar to the observed data. The results provide evidence for temporal structure in our dataset (3).

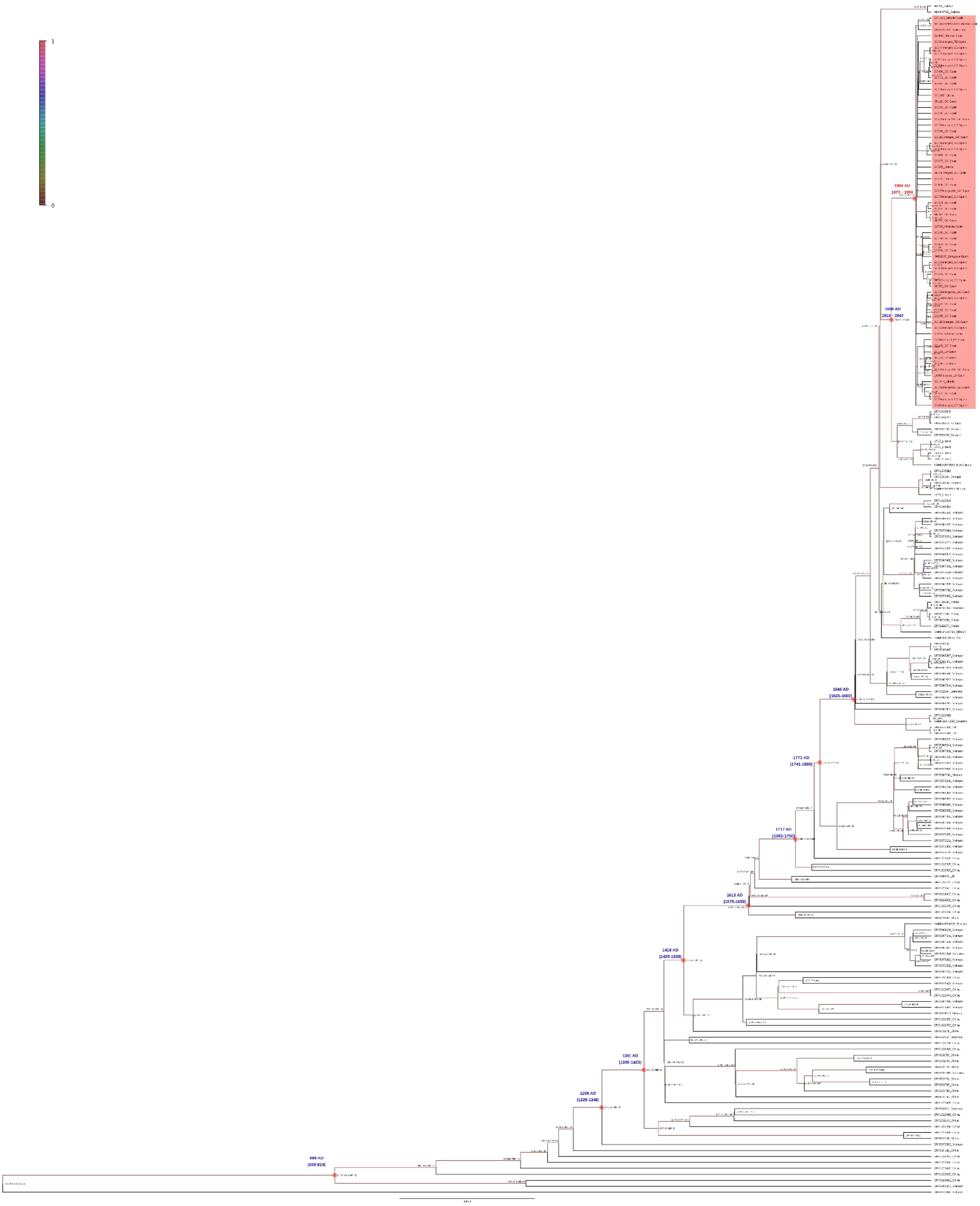

Figure S1. Maximum clade credibility tree obtained with Beast. 95% Highest probability density intervals (95% HPD) are indicated for all nodes. Median time and 95%HPD of the most recent common ancestor of the GC outbreak (in red) and related nodes (in blue), are indicated. Color branch denotes posterior values as indicated in the color scale.

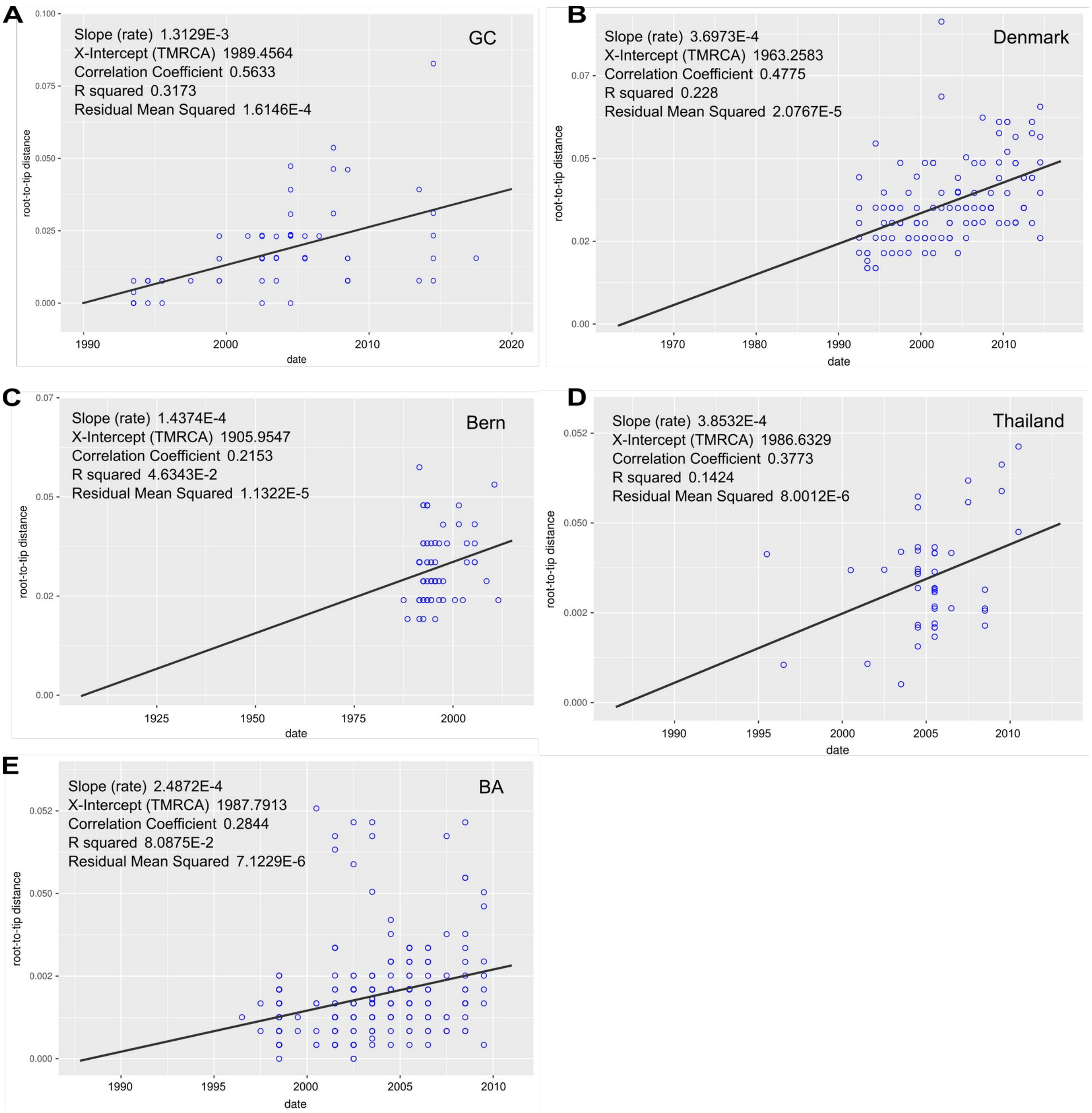

Figure S2. Root to tip analyses obtained with Tempest for GC (A), Denmark (B), Bern (C), Thailand (D) and Buenos Aires (E) outbreaks. Clock rate (slope), time of the most recent common ancestor (TMRCA) and Rsquared are indicated in each plot.

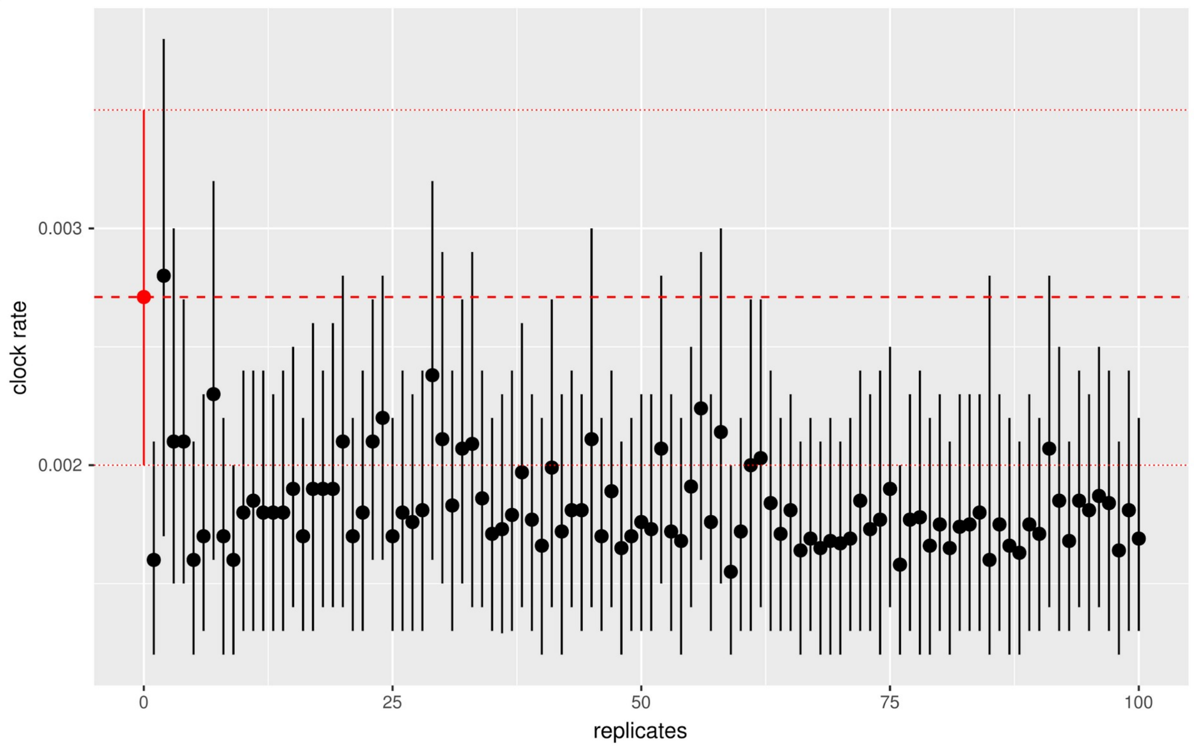

Figure S3. Date randomization test (DRT) performed for GC outbreak. Clock rate with 95% Highest posterior density interval is plotted for each of the 100 replicates with dates randomized. In read the values obtained with the observed data.

References

1. Caminero JA, Pena MJ, Campos-Herrero MI, Rodríguez JC, García I, Cabrera P, Lafoz C, Samper S, Takiff H, Afonso O, Pavón JM, Torres MJ, van Soolingen D, Enarson DA, Martin C. 2001. Epidemiological evidence of the spread of a Mycobacterium tuberculosis strain of the Beijing genotype on Gran Canaria Island. Am J Respir Crit Care Med 164:1165–1170.

2. Pena M, Caminero J, Campos-Herrero M, Rodriguez-Gallego J, Garcia-Laorden M, Cabrera P, Torres M, Lafarga B, Rodriguez D, Samper S, Canas F, Enarson D, Martin C. 2003. Epidemiology of tuberculosis on Gran Canaria: a 4 year population study using traditional and molecular approaches. Thorax 58:618.

3. Folkvardsen DB, Norman A, Andersen ÅB, Michael Rasmussen E, Jelsbak L, Lillebaek T. 2017. Genomic Epidemiology of a Major Mycobacterium tuberculosis Outbreak: Retrospective Cohort Study in a Low-Incidence Setting Using Sparse Time-Series Sampling. J Infect Dis 216:366–374.

4. Stucki D, Ballif M, Bodmer T, Coscolla M, Maurer A-M, Droz S, Butz C, Borrell S, Längle C, Feldmann J, Furrer H, Mordasini C, Helbling P, Rieder HL, Egger M, Gagneux S, Fenner L. 2015. Tracking a tuberculosis outbreak over 21 years: strain-specific single-nucleotide polymorphism typing combined with targeted whole-genome sequencing. J Infect Dis 211:1306–1316.

5. Coscolla M, Barry PM, Oeltmann JE, Koshinsky H, Shaw T, Cilnis M, Posey J, Rose J, Weber T, Fofanov VY, Gagneux S, Kato-Maeda M, Metcalfe JZ. 2015. Genomic epidemiology of multidrug-resistant Mycobacterium tuberculosis during transcontinental spread. J Infect Dis 212:302–310.

6. Eldholm V, Monteserin J, Rieux A, Lopez B, Sobkowiak B, Ritacco V, Balloux F. 2015. Four decades of transmission of a multidrug-resistant Mycobacterium tuberculosis

outbreak strain. Nat Commun 6:7119.
